# Mortality from Circulatory System Diseases and Malformations in Children in the State of Rio de Janeiro

**DOI:** 10.5935/abc.20160069

**Published:** 2016-06

**Authors:** Thais Rocha Salim, Gabriel Porto Soares, Carlos Henrique Klein, Glaucia Maria Moraes de Oliveira

**Affiliations:** 1 Pós-Graduação em Cardiologia - Universidade Federal do Rio de Janeiro, Rio de Janeiro, RJ, Brazil; 2 Instituto de Cardiologia Edson Saad - Universidade Federal do Rio de Janeiro, Rio de Janeiro, RJ, Brazil; 3 Escola Nacional de Saúde Pública da Fundação Oswaldo Cruz, Rio de Janeiro, RJ, Brazil

**Keywords:** Cardiovascular Defects, Heart Defects, Congenital / mortality, Heart Defects, Congenital / epidemiology, Child Mortality

## Abstract

**Background:**

The epidemiological profile of mortality in a population is important for the institution of measures to improve health care and reduce mortality

**Objective:**

To estimate mortality rates and the proportional mortality from cardiovascular diseases and malformations of the circulatory system in children and adolescents.

**Methods:**

This is a descriptive study of mortality from cardiovascular diseases, malformations of the circulatory system, from all causes, ill-defined causes and external causes in children and adolescents in the state of Rio de Janeiro from 1996 to 2012. Populations were obtained from the Brazilian Institute of Geography and Statistics (Instituto Brasileiro de Geografia e Estatística - IBGE) and deaths obtained from the Department of Informatics of the Unified Health System (DATASUS)/Ministry of Health.

**Results:**

There were 115,728 deaths from all causes, 69,757 in males. The annual mortality from cardiovascular diseases was 2.7/100,000 in men and 2.6/100,000 in women. The annual mortality from malformations of the circulatory system was 7.5/100,000 in men and 6.6/100,000 in women. Among the specific causes of circulatory diseases, cardiomyopathies had the highest rates of annual proportional mortality, and from malformations of the circulatory system, it occurred due to unspecified malformations of the circulatory system, at all ages and in both genders.

**Conclusion:**

Mortality from malformations of the circulatory system was most striking in the first years of life, while cardiovascular diseases were more relevant in adolescents. Low access to prenatal diagnosis or at birth probably prevented the proper treatment of malformations of the circulatory system.

## Introduction

In Brazil, in the year 2012, cardiovascular diseases were the leading cause of death in the general population, but children and adolescents do not show that mortality profile.^1^ In children younger than 1 year, cardiovascular diseases are the ninth cause of death; in children aged 1 to 9 years, it is the eighth; in those aged 10 to 14 years, the seventh; and in those aged 15 to 19 years, it is the fourth most important cause of death, considering the chapters of the International Classification of Diseases (ICD-10).^1,2^ External causes (EC) are the main cause of death, except in children younger than 1 year, for whom they occupy the sixth position. As for ill-defined causes (IDC) of mortality, they range between the fifth and seventh positions in those younger than 15 years, being the third most important cause in children aged 15 to 19 years.^1^

Children younger than 1 year have the highest rates of overall mortality, with the main causes of deaths being conditions originating in the perinatal period, which corresponded to 58.6% of deaths in this age group in Brazil in 2012.^1,3^

Child mortality is divided into two components: the neonatal and the post-neonatal period.^3^ Its causal factors are closely linked to health and nutrition, and to women's educational and socioeconomic level, as well as the quality of care provided during prenatal care and delivery, and assistance at birth.^4^ With the improvement of these factors, there was a change in the distribution of infant mortality composition from 1994 to 2012, with a reduction in the post-neonatal period, but little has changed in the neonatal period.^5^

The main causes of neonatal mortality are intrauterine and intrapartum asphyxia, extreme prematurity and congenital malformation.^6,7^ Among the congenital malformations, those of the circulatory system have greater impact on mortality, being classified as preventable causes of death, as they could be reduced by early interventions.^6^ High mortality rates due to malformations of the circulatory system (MCS) result from prenatal diagnosis scarcity, resulting in ineffective treatment, with consequent death.^8^

Few national publications report studies on mortality from circulatory system diseases and MCS in the pediatric population. To know the epidemiological profile of mortality of a population is important to implement improvement measures of assistance in health and reduced mortality.

The aim of this study was to estimate the rates of mortality per inhabitant and proportional mortality from circulatory system diseases and MCS in children and adolescents.

## Methods

Descriptive study of rates of mortality from circulatory system diseases and MCS from all causes (AC), IDC and EC in children and adolescents in the state of Rio de Janeiro from 1996 to 2012. The children were divided into three age groups: children younger than 1 year, children aged 1 to 4 years and 5 to 11 years. Adolescents, as defined in the Statute of Children and Adolescents, are those aged 12 to 17 years.^9,10^

Data related to the deaths were obtained from the Department of Informatics of the Brazilian Unified Health System (DATASUS) (http://tabnet.datasus.gov.br/cgi/sim/dados/cid10_indice.htmdados). These data consist of sets of all death certificates (DC) recorded in the state of Rio de Janeiro, from 1996 to 2012, year by year. From each database of annual data, we selected only the deaths of live births up to the age of full 17 years.^1,11^ During the period, the code of the underlying cause of death was used, according to ICD 10.^2^

Diseases of the circulatory system correspond to deaths of which underlying cause was any one from Chapter IX of ICD-10. The specific causes of death from circulatory diseases were: rheumatic fever (I 00-09); hypertensive diseases (I 10-15); ischemic heart disease (I 20-21); pulmonary heart diseases and pulmonary circulation diseases (I 26-28); membranes (pericarditis I30-I32 and acute and subacute endocarditis I33); valvular diseases (I34-39); myocarditis (I40- 41); cardiomyopathies (I42-43); conduction diseases (I44-49); heart failure (I50); complications of heart disease and ill-defined heart diseases (I51-52); hemorrhagic cerebrovascular diseases (I60-62); cerebral infarction (I63); unspecified cerebrovascular accident (I64); other cerebrovascular diseases (I65-69); vascular diseases (I70-89); and other unspecified diseases of the circulatory system (I95-99).

The deaths of which underlying cause was MCS corresponds to the chapter XVII of the ICD-10 and were discriminated in the categories heart chambers and septal defects (Q20); cardiac septa (Q21); pulmonary and tricuspid valves (Q22); aortic and mitral valves (Q23); others and non-specified (Q24); great arteries (Q25); other vessels (Q26-28). The deaths of which underlying causes were IDC correspond to those of Chapter XVIII of the ICD-10. The deaths from EC are those from Chapters XIX and XX of ICD-10, which were also discriminated into categories: transportation accidents (V01-99), other accidental trauma (W00-X59) and non-accidental external causes (X60-Y98). Deaths were grouped into four categories of time: 1996 to 1999, 2000 to 2004, 2005 to 2009 and 2010 to 2012.

Data related to the populations from 1996 to 2012 were obtained from the DATASUS site (http://tabnet.datasus.gov.br/cgi/deftohtm.exeibge/cnv/poprj.def). These population data were grouped according to age, gender and periods, similarly to the deaths, in order to estimate annual mortality rates per 100,000 inhabitants.

Total proportional mortality was calculated for each group of causes, without exclusions, and from defined endogenous causes, excluding the ill-defined and external causes, in percentages.

Bar charts were built of the annual proportional mortality from specific causes of the circulatory system (cardiovascular diseases and MCS) per age and gender, from 1996 to 2012. The quantitative procedures were performed using the programs Excel-Microsoft^12^ and STATA.^13^

The study was carried out in accordance with the current ethical principles and was approved by the research ethics committee of Hospital Universitário Clementino Fraga Filho, which belongs to Universidade Federal do Rio de Janeiro.

## Results

From 1996 to 2012, there were 115,728 deaths from AC in individuals younger than 18 years in the state of Rio de Janeiro, of which 69,757 were males and 45,971 females. The mean annual mortality from AC was 442.7 per 100,000 in both genders, and 530.6 in the male and 353.8 in the female gender. During the same period, there were 1,986 deaths, of which the underlying cause was classified as cardiovascular diseases, with 1,026 occurring in males and 958 in females. Therefore, the annual mortality from cardiovascular diseases was 2.6 deaths per 100,000 inhabitants in both genders, and 2.7 in males and 2.6 in females. The proportional mortality from circulatory diseases, i.e., the percentage of deaths from this group of causes in relation to total deaths, was 1.7, 1.5 and 2.1%, respectively.

During the same period, there were 5,287 deaths, of which the underlying cause was classified as MCS, with 2,837 occurring in males and 2,450 in females. The annual mortality from MCS was 7.0 per 100,000 in both genders, with 7.5 in males and 6.6 in females, with proportional mortalities of 4.6, 4.1 and 5.3%, respectively.

However, 24,111 deaths were classified as caused by IDC or EC, of which 18,906 in males and 5,205 in females, with a proportional mortality of 20.8, 27.1 and 11.3%, respectively. Excluding the diseases of which the underlying cause was IDC or EC, the proportional mortality from circulatory system diseases was 2.2% in both genders, 2.0% in males and 2.3% in females, whereas the proportional mortality from MCS increased to 5.8% in both genders, 5.6% in males and 6.0% in females. It should also be noted that during the period, there were 12,696 deaths due to malformations in any organ or system, of which 6,719 occurred in males and 5,977 in females. Thus, 41.6% of deaths occurred from MCS, of which 42.2% in males and 41.0% in females.

Results according to age groups can be seen in Tables 1 to 6. The largest mortality rates from AC were observed in males in all age groups, with children younger than 1 year being the group with the highest rates (Table 1). Mortality rates from MCS in boys were higher than in girls in all groups, except for those aged 5 to 11 years. Mortality rates from circulatory system diseases were higher in girls than in boys in the groups of children younger than 1 year and 1 to 4 years and similar in those aged 5 to 11 years; however, in the group of adolescents (12 to 17 years), it was the opposite, with the mortality rate in boys being higher than in girls (Table 1).

**Table 1:** Total Proportional mortality and by defined endogenous causes* and annual mortality per 100,000 in children** and adolescents due to diseases and malformations of the circulatory system, and mortality from all causes, according to gender and age group, in the state of Rio deB Janeiro , 1996-2012

Causes of death		Male				Female			
		Age <1	1-4 years	5-11 years	12-17 years	Age <1	1-4 years	5-11 years	12-17 years
Diseases of the circulatory system	Deaths	233	177	174	442	257	187	174	340
	Total PM (%)	0.6	2.9	3.4	2.4	0.8	3.7	4.6	6.2
	Endogenous PM (%)*	0.6	4.4	6.3	13.5	0.9	5.3	7.3	12.2
	Mortality per 100,000	11.4	2.2	1.2	3.4	13.1	2.4	1.2	2.6
Malformations of the circulatory system	Deaths	2,385	283	95	74	2,038	259	102	51
	Total PM (%)	5.9	4.6	1.9	0.4	6.4	5.2	2.7	0.9
	Endogenous PM (%)*	6.4	7.0	3.5	2.3	6.9	7.3	4.3	1.8
	Mortality per 100,000	116.3	3.5	0.6	0.6	104.0	3.3	0.7	0.4
All causes	Deaths	40,223	6,207	5,144	18,183	31,725	5,020	3,755	5,471
	Mortality per 100,000	1962.1	76.2	35.0	138.3	1619.1	63.8	26.4	42.1

* Excluding the ill-defined and external causes (ICD-10 chapters XVIII to XXII);

** mortality of children younger than 1 year per live births. PM: Proportional mortality.

As for the total proportional mortality from circulatory system diseases and MCS, that is, without excluding IDC or EC, the girls always showed higher rates than the boys' in the four age groups (Table 1). However, only the endogenous proportional mortalities, excluding IDC and the EC, were higher in boys in the age group of the adolescents, mainly due to high mortality rates due to external causes in boys.

When adding the rates of endogenous proportional mortality from diseases of the circulatory system and the MCS, there was a relative increase in the participation of the total, from approximately 7.2% in the first year of life to up to 15.8% in male adolescents. In girls, this progression was 7.8 to 14.0%. When evaluating the total proportional mortality, without excluding IDC and EC, such difference was not observed in males (Table 1).

The differences between total and endogenous proportional mortality from diseases of the circulatory system increased from the younger group to the older group of adolescents, with more emphasis on the male gender, and the opposite occurred with total and endogenous proportional mortalities from MCS (Table 1).

The IDC are more important in the group aged 1 to 4 years, both in boys and girls, which represented just over 10% of all deaths (Table 2). In relation to the EC, transportation accidents were the most important from 5 to 11 years; as for non-accidental external causes, they were extremely relevant in the group of adolescents, especially among boys, in which approximately six in ten deaths were caused, mostly, by injuries and aggression. Even among girls, approximately two out of ten deaths were also caused by this fraction of the EC (Table 2). The fraction of trauma caused by accidents became relevant as early as in the group of 1 to 4 years of age (Table 2).

**Table 2:** Proportional mortality from ill-defined and external causes in children and adolescents, according to gender and age group in the state of Rio de Janeiro, 1996-2012

Causes of death		Male				Female			
		Age <1 year	1-4 years	5-11 years	12-17 years	Age <1 year	1-4 years	5-11 years	12-17 years
Ill-defined	Deaths	1,607	709	296	816	1.248	506	282	488
	PM (%)	4.0	11.4	5.8	4.5	3.9	10.1	7.5	8.9
Transportation accidents	Deaths	70	272	861	1,746	50	211	485	685
	PM (%)	0.2	4.4	16.7	9.6	0.2	4.2	12.9	12.5
Other accidental traumas	Deaths	996	901	872	1,526	740	546	421	353
	PM (%)	2.5	14.5	17.0	8.4	2.3	10.9	11.2	6.5
External non-accidental causes	Deaths	215	264	372	10,811	154	214	192	1,154
	PM (%)	0.5	4.3	7.2	59.5	0.5	4.3	5.1	21.1
All causes	Deaths	40,223	6,207	5,144	18,183	31,725	5,020	3,755	5,.471
	PM (%)	100.0	100.0	100.0	100.0	100.0	100.0	100.0	100.0

PM: prorpotional mortality.

The period of 1996-1999 showed higher AC mortality rates in all age groups in both sexes (Tables 3 to 6). In groups aged 1-4 years, 5-11 years and in adolescents there was a slight increase in AC mortality rates in the 2010-2012 period, when compared with the two previous years in both genders (Table 4). The exception occurred in adolescents, whose last period showed the lowest AC mortality rate (Table 6).

**Table 3:** Total proportional mortality and from defined endogenous causes* and annual mortality per 100,000 live births in the first yearo f life** from diseases and malformations of the circulatory system, and froma ll causes, according to gender and age group, in the state ofR io de Janeiro, 1996-2012

Causes of death		Male				Female			
		1996-1999	2000-2004	2005-2009	2010-2012	1996-1999	2000-2004	2005-2009	2010-2012
Diseases of the circulatory system	Deaths	53	51	77	41	57	69	71	43
	Total PM (%)	0.4	0.5	0.9	0.8	0.5	0.9	1.0	1.1
	Endogenous PM (%)*	0.4	0.6	0.9	0.9	0.6	1.0	1.1	1.2
	Mortality per 100,000 LB	9.7	8.3	13.8	12.2	11.0	11.8	13.3	13.4
Malformations of the circulatory system	Deaths	630	572	645	394	551	517	511	318
	Total PM (%)	4.5	5.8	7.1	7.8	5.1	6.6	7.1	7.8
	Endogenous PM (%)*	4.9	6.2	7.6	8.5	5.5	7.1	7.6	8.5
	Mortality per 100,000 LB	115.7	93.6	115.7	116.9	106.1	88.4	95.8	98.8
All causes	Deaths	13,965	9,915	9,065	5,071	10,839	7,823	7,247	4,072
	Mortality per 100,000 LB	2,563.9	1,622.4	1,626.7	1,504.9	2,087.1	1,337.9	1,358.1	1,265.5

* Excluding the ill-defined and external causes (ICD-10 chapters XVIII to XXII);

** mortality of children younger than 1 year per live births. PM: prorpotional mortality; LB: live births.

**Table 4:** Total proportional mortality and from defined endogenous causes* and annual mortality per 100,000 inhabitants aged 1 to 4 years, from diseases and malformations of the circulatory system, and from all causes, according to gender and age group in the state of Rio de Janeiro 1996-2012

Causes of death		Male				Female			
		1996-1999	2000-2004	2005-2009	2010-2012	1996-1999	2000-2004	2005-2009	2010-2012
Diseases of the circulatory system	Deaths	49	41	52	26	66	35	46	30
	Total PM (%)	2.5	2.7	3.5	2.9	4.0	2.9	3.9	4.5
	Endogenous PM (%)*	3.8	4.2	5.3	4.4	5.6	4.3	5.2	6.2
	Mortality per 100,000	2.7	1.6	2.1	2.2	3.7	1.4	1.9	2.6
Malformations of the circulatory system	Deaths	79	77	80	27	80	64	73	32
	Total PM (%)	4.0	5.1	5.4	3.1	4.8	5.3	6.1	4.8
	Endogenous PM (%)*	6.2	7.9	8.1	4.6	6.8	7.8	8.3	6.7
	Mortality per 100,000	4.3	3.0	3.2	2.2	4.5	2.6	3.0	2.7
All causes	Deaths	1,996	1,519	1,477	884	1,669	1,218	1,192	671
	Mortality per 100,000	107.9	59.5	58.2	73.4	92.8	49.4	48.9	57.3

* Excluding the ill-defined and external causes (ICD-10 chapters XVIII to XXII); PM: prorpotional mortality.

**Table 5:** Total proportional mortality and from defined endogenous causes* and annual mortality per 100,000 inhabitants aged 5 to 11 years, from diseases and malformations of the circulatory system, and from all causes, according to gender and age group in the state of Rio de Janeiro, 1996-2012

Causes of death		Male				Female			
		1996-1999	2000-2004	2005-2009	2010-2012	1996-1999	2000-2004	2005-2009	2010-2012
Diseases of the circulatory system	Deaths	41	35	60	27	48	39	50	26
	Total PM (%)	2.8	2.9	4.3	3.4	4.7	4.7	4.7	4.2
	Endogenous PM (%)*	5.9	5.7	7.2	5.9	8.0	7.5	7.0	6.6
	Mortality per 100,000	1.2	0.8	1.3	1.1	1.5	0.9	1.1	1.1
Malformations of the circulatory system	Deaths	26	23	29	12	30	25	25	15
	Total PM (%)	1.8	1.9	2.1	1.5	2.9	3.0	2.3	2.4
	Endogenous PM (%)*	3.8	3.7	3.5	2.6	5.0	4.8	3.5	3.8
	Mortality per 100,000	0.8	0.5	0.6	0.5	0.9	0.6	0.6	0.6
All causes	Deaths	1,448	1,215	1,405	805	1,030	828	1,071	619
	Mortality per 100,000	43.5	27.9	30.8	32.6	32.0	19.6	24.3	25.9

* Excluding the ill-defined and external causes (ICD-10 chapters XVIII to XXII). PM: prorpotional mortality.

**Table 6:** Total proportional mortality and from defined endogenous causes* and annual mortality per 100,000 inhabitants aged 12 to 17 years, from diseases and malformations of the circulatory system, and from all causes, according to gender and age group in the state of Rio de Janeiro, 1996-2012

Causes of death		Male				Female			
		1996-1999	2000-2004	2005-2009	2010-2012	1996-1999	2000-2004	2005-2009	2010-2012
Diseases of the circulatory system	Deaths	114	97	140	69	128	65	89	43
	Total PM (%)	2.4	2.2	2.6	2.7	8.1	5.1	6.3	4.6
	Endogenous PM (%)*	13.1	14.3	14.9	11.1	16.6	11.1	11.6	8.2
	Mortality per 100,000	3.7	2.5	3.7	2.9	4.1	1.7	2.4	1.8
Malformations of the circulatory system	Deaths	18	19	20	14	12	14	11	10
	Total PM (%)	0.4	0.4	0.4	0.6	0.8	1.1	0.8	1.1
	Endogenous PM (%)*	2.1	2.8	2.1	2.3	1.6	2.4	1.4	1.9
	Mortality per 100,000	0.6	0.5	0.5	0.6	0.4	0.4	0.3	0.4
All causes	Deaths	4.849	4.389	5.354	2.520	1.587	1.268	1.418	926
	Mortality per 100,000	155.6	115.1	139.6	105.9	51.0	33.5	37.6	39.7

* Excluding the ill-defined and external causes (ICD-10 chapters XVIII to XXII). PM: prorpotional mortality.

Mortality rates from cardiovascular diseases increased significantly only in children younger than 1 year, most evidently in children in the last two periods, compared to the first two (Table 3). In other age groups, there were minor fluctuations, except in girls aged 1 to 4 years and in adolescents, in whom, in the first period (1996-1999), mortality rates from cardiovascular diseases were higher (Tables 4 to 6).

Mortality rates from MCS exceeded more than one death per thousand births in children younger than 1 year in both genders (Table 1). In all study periods, MCS mortality rates in boys were higher than in girls. At the age range 1 to 4 years, MCS mortality rates were even higher than for circulatory system diseases (Table 4). In the two older age groups, those rates are quite low, and all represent less than one death per 100,000 individuals (Tables 5 and 6).

**Figure 1:**
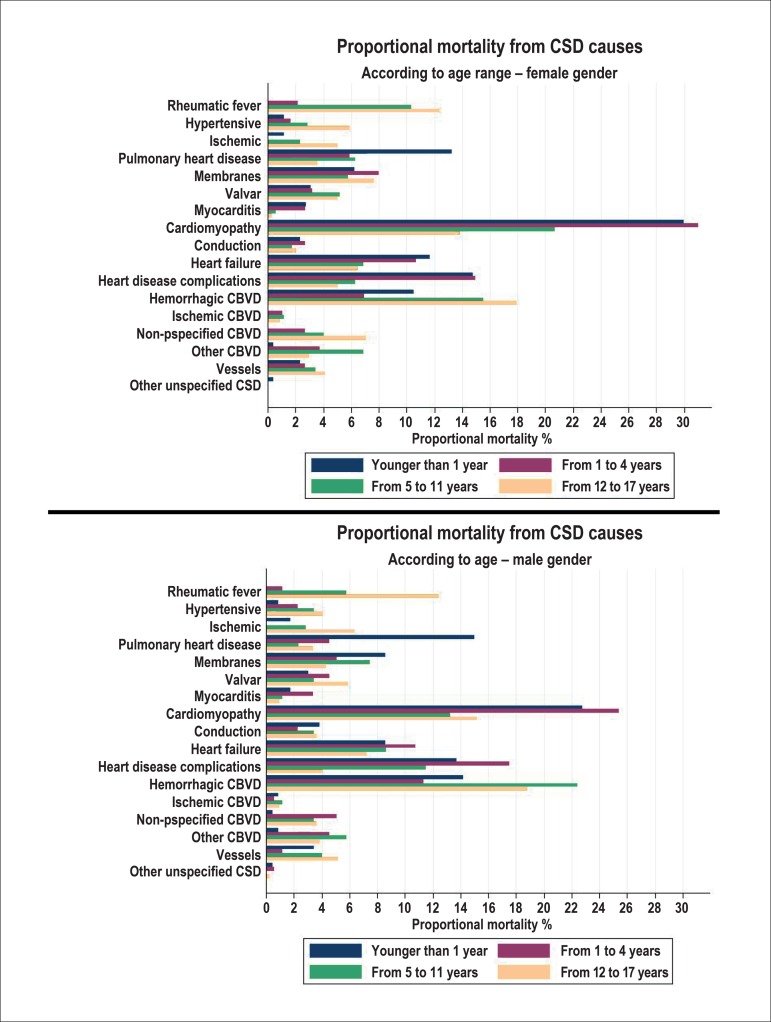
Annual proportional mortality from specific causes of the circulatory system in children and adolescents, by gender and age group in the state of Rio de Janeiro, from 1996 to 2012. CSD: circulatory system diseases; CBVD: cerebrovascular disease.

Among the specific causes of cardiovascular diseases, cardiomyopathies showed the highest rates of annual proportional mortality in both genders (Figure 1). Cardiomyopathies were predominant mainly in the age group 1 to 4 years (25.4% in boys and 31% for girls). Cardiomyopathies were followed by hemorrhagic cerebrovascular disease, with predominance in the age range 5 to 11 years for males and 12 to 17 years in females. Heart disease complications were in the third place, with a higher percentage in children younger than 1 year and aged 1 to 4 years, with a slight predominance of females. Pulmonary heart and pulmonary circulation diseases came after that, in both genders, in children younger than 1 year, followed by rheumatic fever at the age range of 12 to 17 and 5 to 11 years (Figure 1).

**Figure 2:**
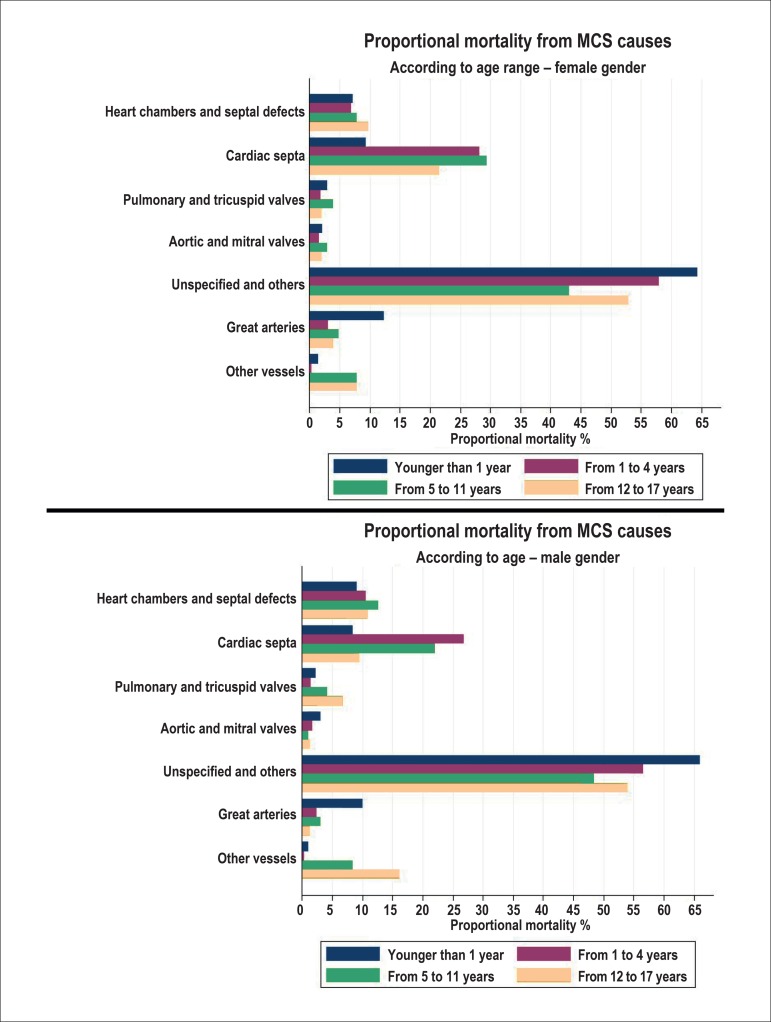
Annual proportional mortality from specific causes of malformations of the circulatory system in children and adolescents, by gender and age group in the state of Rio de Janeiro, from 1996 to 2012. MCS: malformations of the circulatory system.

The greatest proportional mortality from MCS occurred from unspecified MCS according to ICD 10, at all ages and in both genders. They were followed by cardiac septal defects, except in the group of children younger than 1 year in both genders, in whom malformations of the great arteries came next. In adolescent males, the second position was occupied by malformations of other vessels (Figure 2).

## Discussion

Diseases of the circulatory system are the leading cause of adult mortality, especially those secondary to atherosclerotic diseases, such as cerebrovascular disease and ischemic heart disease, which together accounted for over 60% of deaths from cardiovascular diseases in the state of Rio de Janeiro in 2012.^14^ In children younger than 1 year of age, deaths due to malformations are among the leading causes of death, second only to perinatal conditions.

Among the malformations, those of the circulatory system are the main components.^1^ The MCS contribute mostly to the deaths in children younger than 1 year and aged 1 to 4 years, because these causes are often incompatible with life and highly dependent on adequate hospital-medical support for survival, leading to early mortality, with progressive reduction in the following age groups. This behavior is opposite to that of circulatory diseases, which show progressive increase until they become the leading cause of death in adults after the fifth decade of life.^1^

In adolescents of both genders, when analyzing the endogenous proportional mortality, it can be observed that diseases of the circulatory system, from that age group on, are among the leading causes of death. However, at this age range, the major cause of death from circulatory diseases is cardiomyopathy, not atherosclerotic disease, which occurs predominantly at older ages.^15^

Among the MCS, unspecified diseases are the leading cause of death at all age ranges and in both genders, suggesting low access to prenatal diagnosis or at birth, preventing adequate treatment, resulting in death. Some studies show that 30% of MCS are not diagnosed within the first week of life.^16,17^ Measures such as prenatal care and performing obstetric echocardiography could reduce these deaths, allowing early diagnosis and patient referral to specialized treatment centers, even before birth.^17^ However, a major problem constitutes the service network of public and private health systems, not only in the state of Rio de Janeiro, but in the whole country, which cannot treat 62% of children with MCS, reaching in some regions of Brazil , 76 to 91% of cases^16,18^.

If one considers that the MCS can be treated, classifying them in the category of preventable deaths, the appropriate care of the pediatric population would result in significant decrease in child mortality rates, mainly in the early neonatal period.^19,20^ Additionally, the early treatment of children with congenital heart disease prevents successive hospital admissions for complications of the disease and ensures better quality of life.^21,22^

It is observed that mortality from circulatory diseases decreases up to the age range of 5 to 11 years, increasing again in adolescents, whereas in MCS, the decrease is continuous and much more pronounced. It must be considered that children with MCS, often already repaired and that did not die in the first year of life, can have complications and sequelae such as heart failure, arrhythmias, endocarditis, among others, which can lead to death in adolescence, increasing mortality from circulatory system diseases in this age group.^23^

The complications of heart disease, which are the third cause of circulatory system diseases and mean acquired structural heart complications due to residual disease from previous surgical treatments such as chordae tendineae or papillary muscle rupture, intracardiac thrombosis, cardiomegaly, acquired septal defect and other ill-defined or unspecified heart diseases increase the occurrence of death from circulatory system diseases.

The total proportional mortality from circulatory system diseases in males does not increase as the age groups advance. This finding is related to what is observed in the proportional mortality from IDC and external causes, especially in the group of the latter related to non-accidental EC, mainly comprising injuries and aggression, as the impact of mortality from IDC and EC in boys is observed mainly in adolescents. In females, the participation of the sum of mortalities is constant throughout childhood and adolescence, when IDC and EC are not excluded. The predominance of the male gender in mortality from EC found in this study has been observed in many parts of the world.^24,25^

This fact is explained by the male gender's greater exposure to risk factors, such as alcohol, tobacco or other drugs, use of firearms or knives, school absenteeism and inclusion in activities considered illicit.^26^ As for the fact that intentionality changes according to age, with a prevalence of accidents among children and violence among adolescents, one must consider that children are more exposed to accidents caused by their own immaturity, curiosity, intense growth and development, resulting in a higher proportion of accidental causes, especially in the domestic environment.^27^ Whereas adolescents are more vulnerable to violence, because of social marginality and drug exposure, among other negative events.^28,29^

In the period from 2010 to 2012, the mortality rate from AC in male adolescents showed a relevant decrease of 24% compared to previous periods, which did not occur in females. During the same period, in the state of Rio de Janeiro, there was a decrease in deaths from homicides caused by the police, recorded in the so-called acts of resistance to authority, in which the main victims are male adolescents and young adults.^30^ This fact may be related to this significant reduction in the mortality rate.

The limitation of this study was the quality variation in the filling-out of death certificates (DCs) over time and study location, the state of Rio de Janeiro. However, the DCs are the best available sources of mortality data.

## Conclusion

In the state of Rio de Janeiro, from 1996 to 2012, there was a progressive reduction in mortality from all causes in children and adolescents. The highest mortality rates were observed in children younger than 1 year and in the male gender. Mortality from malformations of the circulatory system was higher in the early years, while mortality from cardiovascular diseases became more important in adolescents. There is a difference in the mortality profile between the genders, as the boys died more frequently from external causes and girls died mainly from endogenous diseases. Deaths from circulatory system diseases became relevant in male adolescents after deaths from external causes were excluded, predominantly those caused by violence.
